# Global, regional, and national analyses of the burden among adult women of breast cancer attributable to diet high in red meat from 1990 to 2021: longitudinal observational study

**DOI:** 10.3389/fpubh.2025.1580177

**Published:** 2025-05-13

**Authors:** Xiaoyun Ding, Zhenning Tang, Hang Ma, Can Jiang

**Affiliations:** ^1^General Hospital of Ningxia Medical University, Yinchuan, China; ^2^The Department of Anesthesiology, Shanghai Sixth People’s Hospital Affiliated to Shanghai Jiao Tong University School of Medicine, Shanghai, China

**Keywords:** breast cancer, red meat, global burden disease, risk factor, mortality rate

## Abstract

**Background:**

The association between red meat consumption and breast cancer risk has been well established; however, it is crucial to understand the temporal trends, geographical variations, and socio-demographic factors that influence this risk among women aged 25–45. Consequently, this study seeks to investigate the impact of red meat consumption on breast cancer risk among adult women for the first time.

**Methods:**

Data were extracted from the Global Burden of Disease (GBD) 2021 study to calculate age-standardized rates (ASR) for mortality and disability-adjusted life years (DALYs). Trends were assessed using estimated annual percentage change (EAPC) with linear regression analysis. Hierarchical clustering identified temporal trends, and examined the relationships between EAPC, ASR, DALYs, and the socio-demographic index (SDI).

**Results:**

Our findings indicate that breast cancer-related deaths and DALYs attributable to high red meat consumption increased globally, rising from 44,492 deaths and 1,379,721 DALYs in 1990 to 79,956 deaths and 2,407,092 DALYs in 2021. In high SDI regions, age-standardized mortality (−1.47%) and DALYs (−1.48%) rates declined, while low-middle and high-middle SDI regions showed significant increases. Low SDI regions, despite lower absolute numbers, experienced sharp relative increases in both deaths and DALYs. Additionally, a nonlinear relationship between ASR and SDI was observed, with the burden peaking in moderate SDI regions.

**Conclusion:**

This study concludes the rising global burden of breast cancer in adult women associated with high red meat consumption, with particularly pronounced impacts in low and middle SDI regions.

## Graphical abstract

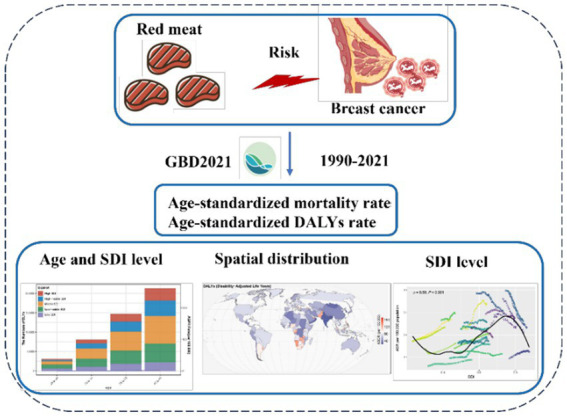

## 1 Introduction

Meat is a vital component of the human diet, offering high-quality protein and essential nutrients, but its rising global consumption raises concerns about potential health impacts. Per capita meat consumption has nearly doubled globally since 1961, with remarkable increases in developing regions, such as China, reflecting improved living standards (1). However, studies suggest that high red meat consumption (RMC) may negatively affect health, with some linking it to an increased risk of cancer (1). While the International Agency for Research on Cancer classified red meat as a Group 2A carcinogen in 2015, indicating it is “probably carcinogenic to humans” (2). The association remains controversial due to inconsistent evidence and significant uncertainty. This debate underscores the need for further research into the health implications of RMC, particularly its possible role in breast cancer, which has emerged as the leading cause of cancer incidence among females globally, with approximately 2.3 million new cases and 670,000 deaths reported in 2022 (3). An estimated 310,720 new cases (32% of total new cases) and 42,250 deaths (15% of the total deaths) of invasive breast cancer are expected to be diagnosed in women in 2024 (4). More than 3 million women are expected to be diagnosed with breast cancer annually by 2040 (5). The incidence and mortality rates vary considerably by region. Despite the rising incidence, the global survival rate for breast cancer has improved over the past three decades. Developed countries have seen a notable 40% reduction in mortality rates over the last 30 years, while underdeveloped nations have made little progress (6).

The Global Burden of Disease (GBD) study has been estimating various disease metrics each year since 1990, offering a valuable opportunity to assess the burden and trends of breast cancer in a comparable way across global, regional, and national levels (7). In addition, the GBD study also estimates the population attributable fractions of risk factors related to breast cancer that may be useful for policy makers to identify intervention priorities for public health actions. Recently, the GBD study 2021, a comprehensive update of epidemiology levels, has incorporated new datasets, enhanced method performance and standardization, and reflected developments in scientific understanding. Nevertheless, there has been no study analyzing the breast cancer burden among adult women attributable to diet high in red meat and trends based on the GBD study 2021 until now. This study aims to provide an in-depth examination of breast cancer disability-adjusted life years (DALYs), deaths, age-standardized-mortality rates (ASMR), and age-standardized DALYs rates (ASDR), with an emphasis on the adult women population that has a higher consumption of diets rich in red meat. By examining these trends, we aim to shed light on actionable strategies that can empower adult women to make informed dietary choices that may mitigate their cancer risk.

## 2 Materials and methods

### 2.1 Data source

The GBD 2021 study provides an extensive assessment of health impacts associated with 369 diseases, injuries, and disabilities, as well as 88 risk factors, across 204 countries and regions, using the most up-to-date epidemiological data and improved standardized methodologies (8). The GBD database utilizes advanced techniques to handle missing data and account for confounding variables. Comprehensive descriptions of the study design and methodologies used in GBD studies are available in existing GBD publications (8). Additionally, the University of Washington Institutional Review Board granted a waiver for informed consent to access GBD data (8, 9). This research followed the Guidelines for Accurate and Transparent Health Estimates Reporting (GATHER) (10). Extracted estimates of deaths and DALYs across different causes, ages, all years, and locations from the GBD 2021 website.^1^ Furthermore, this study used the GBD 2021 estimates that stratified to ages 25–45 years. One study (11) refers to this age group as “later adulthood,” while another defines ages 22–34 as “early adulthood” and ages 35–44 as ‘early middle age” (12). However, a recent study (13) identifies this age group as the “adult women” category, a term we have adopted and consistently used throughout the manuscript.

According to the EAT-Lancet Commission, in a healthy and sustainable diet, red meat intake should be limited to 98 grams per week (~14 grams per day).^2^ High consumption of red meat in the diet was defined as a daily intake exceeding 23 g, including beef, pork, lamb, and goat, but excluding poultry, fish, eggs, and processed meats. The range for high intake levels varied between 18 g and 27 g (14). Therefore, our study classified the use of >23 grams/day as “high intake” which is based on global dietary data and health risk assessments. It is also consistent with the methodology of the GBD study (8). More comprehensive details regarding the criteria for inclusion and exclusion can be found here (9). This study focuses on evaluating the deaths and DALYs burden of breast cancer, while also examining the connection between social demographic factors and a diet rich in red meat.

### 2.2 Definitions

DALY represents the total number of healthy life-years lost from the onset of a disease to death. The age-standardized rate (ASR) is calculated using the age distribution of a standard population. Since overall population mortality and DALY rates are influenced not only by the mortality and DALY rates within specific age groups but also by the age structure of the population, the ASR helps to remove the influence of varying age distributions. This allows for more accurate comparisons of mortality and DALY rates across different regions and time periods.

Additionally, the relationship between disease burden and the socio-demographic index (SDI) was examined. The SDI is a composite measure that reflects the average income per person, fertility rates, and educational attainment in each country or region. The 204 countries and regions were classified into five categories based on their SDI scores: low (SDI < 0.45), low-middle (SDI between 0.45 and 0.61), middle (SDI between 0.61 and 0.69), high-middle (SDI between 0.69 and 0.80), and high (SDI ≥ 0.80) (15).

### 2.3 Statistical analysis

The impact of a diet high in red meat on breast cancer was analyzed by examining several factors such as SDI, region, country, gender, and age group, using measures like DALYs, ASDR, deaths, and ASMR. The ASR was calculated using the following formula:


$$\text{ASR}=\frac{\Sigma_{i=1}^{A}\text{ai}\;\text{wi}}{\Sigma_{i=1}^{A}\text{wi}}$$

where ai represents the age-specific rate in the i th age group, w stands for the number of individuals (or weight) in the corresponding age group from a chosen standard population, and A refers to the total number of age groups. All rates were standardized per 100,000 people to minimize any effects related to population age structure. Instead of utilizing the global population size for 2021, the global standard population from the GBD 2021 study was applied as the weight.

Additionally, linear regression analysis was employed to calculate the EAPC, which estimates the annual rate of change over a defined period, serving to assess trends in the ASR. The linear regression model used for this purpose was y=α+βx+ε, where x represents the calendar year, y corresponds to the natural logarithm of ASMR or ASDR, ε is the error term, and β represents the upward or downward trend in ASR.

The EAPC was calculated using the formula: *EAPC = 100 (exp(β) − 1)*. A 95% confidence interval (CI) was derived from the regression model. When both the EAPC and 95% CI were above zero, the ASR was deemed to be on the rise; if they were below zero, a decreasing trend was observed. If neither condition was met, the ASR was considered stable.

The 204 countries and territories were further categorized into four distinct groups using hierarchical cluster analysis, according to observed temporal trends: stable, minor increase, significant increase, or decline. All statistical analyses were conducted using R software version 4.2.3,^3^ with a *p*-value of less than 0.05 considered statistically significant.

## 3 Results

### 3.1 Global trends of breast cancer are attributable to diet high in red meat

In 2021, the global burden of breast cancer attributable to a diet high in red meat was significant, with 79,956 (95% UI = 17,207–25,280) breast cancer-related deaths and 2,407,092 (95% UI = 513,404–782,020) DALYs (Disability-Adjusted Life Years). This represents a marked increase from 1990, which saw 44,492 (95% UI = 9,518–13,160) deaths and 1,379,721 (95% UI = 296,544–431,200) DALYs. However, the pattern of change varied significantly across regions and SDI levels (Table 1).

**Table 1:** Breast cancer DALYs and deaths attributable to red meat consumption.

	1990				2021				EAPC (1990–2021)	
Regions	Death (95% UI)	ASMR/100,000 (95% UI)	DALYs (95% UI)	ASDR/100,000 (95% UI)	Death (95% UI)	ASMR/100,000 (95% UI)	DALYs (95% UI)	ASDR/100,000 (95% UI)	ASMR (95% CI)	ASDR (95% CI)
Global	44491.95 (95187–13.16)	2.11 (5–0)	1379721.38 (2965446–431.2)	63.22 (136–0.02)	79956.96 (172077–25.28)	1.76 (4–0)	2407092.26 (5134048–782.02)	53.95 (115–0.02)	−0.73 (−0.78–0.68)	−0.65 (−0.71–0.6)
SDI region										
High SDI	19641.48 (41857–6.73)	3.22 (7–0)	550636.96 (1170313–219.8)	98 (208–0.04)	23348.5 (50141–9)	2.1 (4–0)	589189.75 (1245368–261.97)	63.6 (134–0.03)	−1.47 (−1.51–1.42)	−1.48 (−1.51–1.44)
High-middle SDI	12663.32 (27065–4.25)	2.32 (5–0)	396201.58 (840158–139.66)	72.57 (154–0.03)	19670.91 (41975–8.13)	1.87 (4–0)	563809.73 (1182859–242.65)	57.01 (120–0.02)	−0.89 (−1–0.79)	−0.99 (−1.08–0.9)
Middle SDI	7590.36 (16477–2.1)	1.35 (3–0)	269085.51 (587587–75.57)	43.61 (95–0.01)	20985.04 (45578–5.7)	1.47 (3–0)	694940.28 (1499961–194.17)	47.74 (103–0.01)	0.11 (0.05–0.17)	0.14 (0.07–0.2)
Low-middle SDI	3018.45 (6561–0.69)	0.92 (2–0)	107963.78 (232548–24.82)	29.58 (64–0.01)	11206.53 (24428–2.84)	1.41 (3–0)	389844.85 (850589–106.39)	46.1 (100–0.01)	1.39 (1.36–1.42)	1.42 (1.39–1.46)
Low SDI	1511.33 (3307–0.32)	1.24 (3–0)	53808.75 (117614–12.81)	39.14 (85–0.01)	4638.64 (10012–1.14)	1.62 (4–0)	166347.34 (358311–43.76)	50.21 (108–0.01)	0.81 (0.69–0.93)	0.75 (0.63–0.87)
GBD region										
Andean Latin America	178.05 (385–0.08)	1.58 (3–0)	6079.64 (13192–3.04)	49.79 (108–0.02)	543.59 (1199–0.18)	1.72 (4–0)	17173.72 (37444–5.86)	53.09 (115–0.02)	0.07 (−0.04–0.19)	0 (−0.12–0.11)
Australasia	446.25 (954–0.27)	3.61 (8–0)	12909.57 (27739–8.87)	110.39 (237–0.07)	590.73 (1256–0.42)	2.12 (4–0)	15344.04 (32319–9.31)	63.79 (135–0.04)	−1.78 (−1.85–1.72)	−1.85 (−1.9–1.8)
Caribbean	341.19 (726–0.17)	2.53 (5–0)	10821.38 (23212–5.65)	77.16 (165–0.04)	731.02 (1605–0.26)	2.56 (6–0)	21476.57 (47250–8.09)	77.81 (171–0.03)	0.16 (0.09–0.23)	0.13 (0.08–0.18)
Central Asia	701.68 (1480–0.33)	2.54 (5–0)	23413.51 (49718–11.11)	84.07 (178–0.04)	899.84 (1955–0.44)	1.88 (4–0)	29693.35 (64029–14.7)	59.2 (128–0.03)	−0.71 (−0.79–0.62)	−0.99 (−1.07–0.9)
Central Europe	2387.14 (5083–0.81)	2.93 (6–0)	70514.29 (149091–23.61)	89.23 (189–0.03)	3395.74 (7232–1.46)	2.77 (6–0)	82138.69 (175622–35.8)	77.55 (166–0.03)	−0.35 (−0.44–0.26)	−0.59 (−0.69–0.5)
Central Latin America	752.48 (1606–0.27)	1.64 (3–0)	26017.64 (55309–8.78)	50.75 (108–0.02)	2610.7 (5570–1.12)	1.9 (4–0)	85300.67 (181065–35.74)	60.96 (129–0.03)	0.37 (0.26–0.48)	0.45 (0.34–0.55)
Central Sub-Saharan Africa	180.04 (408–0.02)	1.37 (3–0)	6468.63 (14776–0.83)	42.8 (97–0.01)	550.32 (1292–0.12)	1.62 (4–0)	19930.72 (47624–4.34)	50.83 (120–0.01)	0.67 (0.37–0.97)	0.68 (0.38–0.97)
East Asia	5692.81 (12509–1.71)	1.22 (3–0)	206087.44 (453328–65.86)	40.93 (90–0.01)	12715.17 (27999–6.59)	1.14 (3–0)	420704.48 (930771–239.84)	38.99 (87–0.02)	−0.51 (−0.64–0.38)	−0.43 (−0.54–0.32)
Eastern Europe	4049.35 (8601–1.31)	2.44 (5–0)	127391.24 (272128–38.55)	81.71 (175–0.03)	4887.89 (10373–1.95)	2.37 (5–0)	134043.69 (287713–52.17)	72.1 (156–0.03)	−0.56 (−0.78–0.33)	−0.9 (−1.12–0.68)
Eastern Sub-Saharan Africa	701.4 (1567–0.11)	1.71 (4–0)	25147.78 (56678–4.34)	52.78 (119–0.01)	2301.88 (5077–0.57)	2.34 (5–0)	82395.24 (182357–22.65)	70.11 (155–0.02)	1.01 (0.9–1.11)	0.88 (0.77–0.98)
High-income Asia Pacific	1044.07 (2208–0.42)	0.95 (2–0)	38113.84 (81064–16.22)	35.42 (75–0.02)	2666.41 (5760–0.76)	1.24 (3–0)	72260.95 (156002–23.25)	43.04 (92–0.02)	0.85 (0.71–0.99)	0.6 (0.44–0.77)
High-income North America	7327.25 (15651–3.12)	3.84 (8–0)	211401.3 (451160–95.46)	120.92 (258–0.06)	8058.33 (17336–3.61)	2.33 (5–0)	212,711 (456938–97.47)	70.41 (151–0.03)	−1.74 (−1.8–1.68)	−1.86 (−1.92–1.8)
North Africa and Middle East	888.2 (1882–0.32)	0.96 (2–0)	33115.43 (70903–13.32)	32.52 (69–0.01)	3823.38 (8144–1.25)	1.57 (3–0)	138525.14 (295256–46.13)	51.35 (109–0.02)	2.1 (1.88–2.32)	1.9 (1.7–2.1)
Oceania	42.97 (93–0.01)	2.64 (6–0)	1628.54 (3547–0.55)	85.65 (186–0.03)	126.36 (278–0.04)	2.95 (7–0)	4750.5 (10467–1.45)	95.65 (211–0.03)	0.38 (0.3–0.46)	0.37 (0.28–0.45)
South Asia	1852.25 (4062–0.22)	0.61 (1–0)	66673.37 (145715–7.97)	19.54 (43–0)	6982.36 (15476–0.82)	0.88 (2–0)	242180.53 (537130–31.51)	28.63 (63–0)	0.99 (0.84–1.14)	1.04 (0.89–1.19)
Southeast Asia	2102.66 (4644–0.37)	1.39 (3–0)	77104.29 (170910–14.37)	46.84 (104–0.01)	7551.42 (16956–1.94)	2.04 (5–0)	259332.72 (583816–67.09)	67.03 (151–0.02)	1.21 (1.15–1.26)	1.13 (1.08–1.18)
Southern Latin America	968.76 (2065–0.71)	3.84 (8–0)	27688.51 (59154–16.6)	110.98 (237–0.06)	1361.14 (2947–0.83)	2.81 (6–0)	35536.4 (76052–24.24)	79.69 (170–0.06)	−0.95 (−1.06–0.83)	−1.02 (−1.1–0.93)
Southern Sub-Saharan Africa	361.79 (805–0.14)	2.29 (5–0)	12055.93 (26569–4.85)	69.37 (153–0.03)	1134.82 (2469–0.36)	3.36 (7–0)	36056.79 (79136–13.54)	98.35 (215–0.04)	1.7 (1.47–1.92)	1.72 (1.45–1.99)
Tropical Latin America	1150.33 (2448–0.42)	2.27 (5–0)	38349.52 (81163–14.7)	69.21 (147–0.03)	3277.99 (7037–1.93)	2.32 (5–0)	101867.32 (217408–57.81)	72.51 (155–0.04)	−0.13 (−0.19–0.06)	−0.09 (−0.15–0.02)
Western Europe	12495.91 (26670–4.06)	3.98 (8–0)	331109.16 (698907–124.1)	119.36 (252–0.05)	12680.5 (27192–4.92)	2.46 (5–0)	290191.04 (623535–131.26)	70.86 (152–0.04)	−1.62 (−1.66–1.57)	−1.74 (−1.79–1.69)

High SDI regions experienced a reduction in the estimated annual percentage change (EAPC) of both ASMR and age-standardized DALYs (ASDR), with decreases of-1.47% (95% CI = −1.51 to −1.42) and −1.48% (95% CI = −1.51 to −1.44), respectively. In contrast, high-middle and low-middle SDI regions showed an increase in EAPC of ASMR and ASDR, particularly in high-middle SDI areas, where the ASMR reached 2.29 per 100,000 (95% CI = 1.41–3.17) in 2021. In low SDI regions, while the overall burden of breast cancer remained relatively low, both deaths and DALYs saw substantial increases. In 2021, these regions reported 4,638 (95% UI = 10,012–1,140) deaths and 166,347 (95% UI = 358,311–43,760) DALYs. Notably, Southern Sub-Saharan Africa showed one of the highest percentage increases in ASMR and ASDR (EAPC), rising by 1.7 (95% CI = 1.47–1.92) and 1.72 (95% CI = 1.45–1.99) per 100,000, respectively, reflecting a shift in dietary habits and increased exposure to risk factors over time (Table 1).

Geographically, East Asia had the highest number of breast cancer-related deaths in 2021, with 12,715 (95% UI = 27,999–6,590) deaths and 420,704 (95% UI = 930,771–239,840) DALYs, representing a significant increase from 1990. In contrast, Australasia showed the lowest rates, with substantial reductions in ASMR and ASDR (EAPC), decreasing by-4.1 (95% CI = −4.54 to −3.65) and-4.58 (95% CI = −5.09 to −4.08), respectively. These changes highlight the regional disparities in the burden of breast cancer, which is attributable to dietary risks.

**Figure 1:**
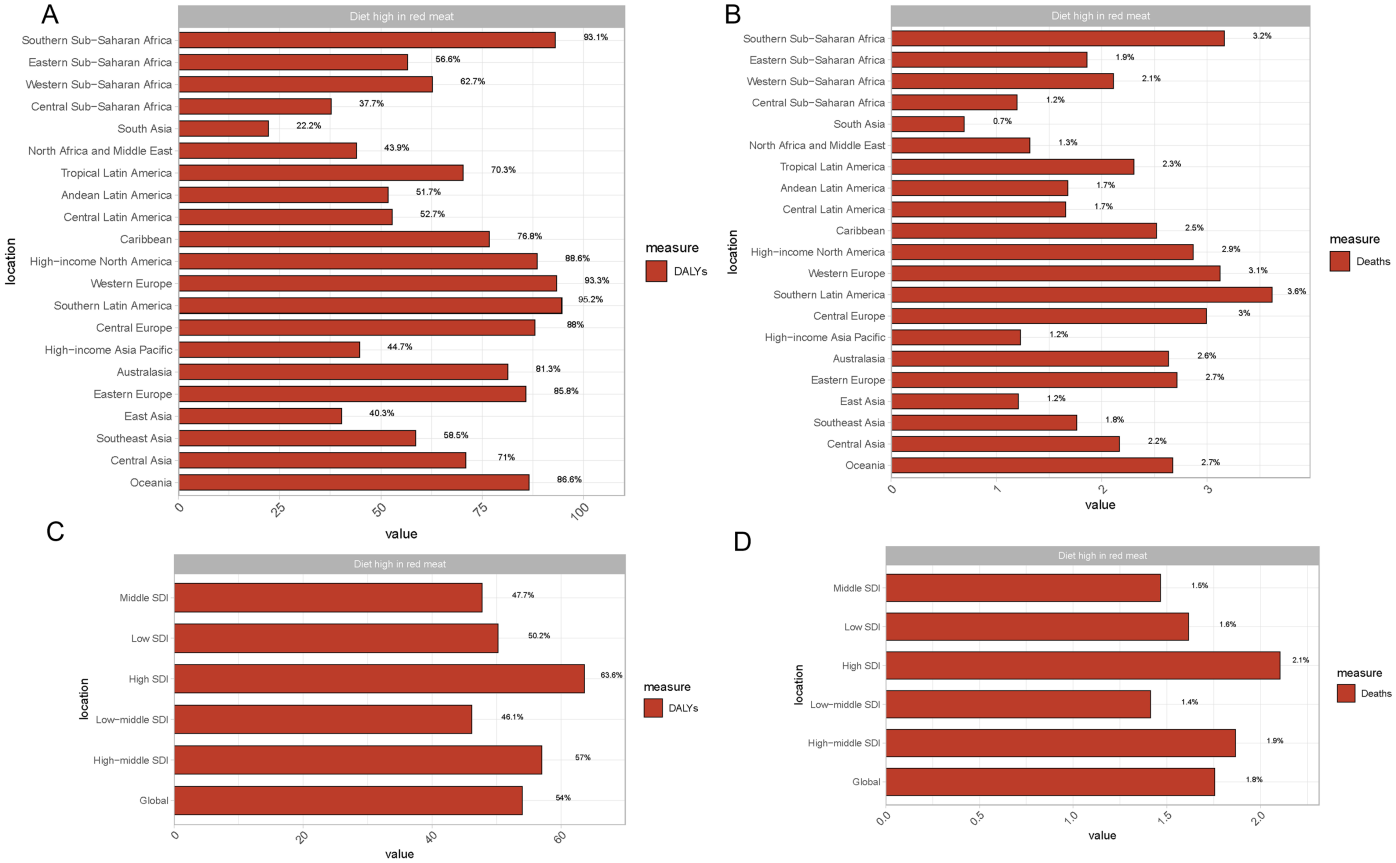
Impact of red meat consumption on DALYs and deaths across regions and SDI levels. **(A)** Proportion of breast cancer DALYs (Disability-Adjusted Life Years) is attributed to diets high in red meat across different global regions. **(B)** Proportion of breast cancer deaths attributed to diets high in red meat across different global regions. **(C)** Proportion of breast cancer DALYs attributed to diets high in red meat across various Sociodemographic Index (SDI) levels. **(D)** Proportion of breast cancer deaths attributed to diets high in red meat across various Sociodemographic Index (SDI) levels.

In both 1990 and 2021, regions such as Central Sub-Saharan Africa, Southeast Asia, and South Asia exhibited relatively high percentages of DALYs attributed to a diet high in red meat. However, the highest percentage of deaths and DALYs in 1990 and 2021 were concentrated in high-income regions such as Oceania and High-income North America, which also show an increasing trend. Conversely, regions like Eastern Europe and Central Asia demonstrated lower percentages of deaths and DALYs, which are attributed to this dietary factor. Notably, the trends highlight a growing disparity between regions, with high-income areas experiencing a higher proportion of diet-related health impacts despite improvements in diet and healthcare overtime (Figure 1).

### 3.2 Country-level breast cancer burden attributable to high red meat consumption

In 2021, the burden of breast cancer attributable to high red meat consumption varied significantly across the globe. According to the data, China reported the highest number of breast cancer deaths, with 125.86 deaths (95% UI: 366–0.04), a significant increase from 44.98 deaths (95% UI: 133–0.02) in 1990. This sharp rise reflects the growing impact of red meat consumption on breast cancer incidence in China over the past three decades. Additionally, China recorded 5,009.78 DALYs (95% UI: 14,867–1.62), indicating a considerable long-term public health impact of breast cancer, which has worsened compared to 1990 (Supplementary Tables S1, S2).

Other countries also face significant breast cancer burdens related to high red meat consumption. The United States reported 30.27 deaths (95% UI: 67–0.02) and 824.35 DALYs (95% UI: 1,821–0.49), while India showed even more severe numbers, with 192.1 deaths (95% UI: 438–0.04) and 6,868.25 DALYs (95% UI: 15,808–1.6). These figures highlight the challenges these countries face in preventing and controlling diet-related cancer risks.

From the perspective of ASDR, smaller island nations such as American Samoa (167.25 per 100,000), Nauru (161.33 per 100,000), and Palau (161.32 per 100,000) exhibited especially high breast cancer burdens. The elevated ASDRs in these countries may indicate greater sensitivity to red meat consumption or limitations in early cancer detection and treatment, leading to higher mortality rates (Supplementary Tables S3, S4).

**Figure 2:**
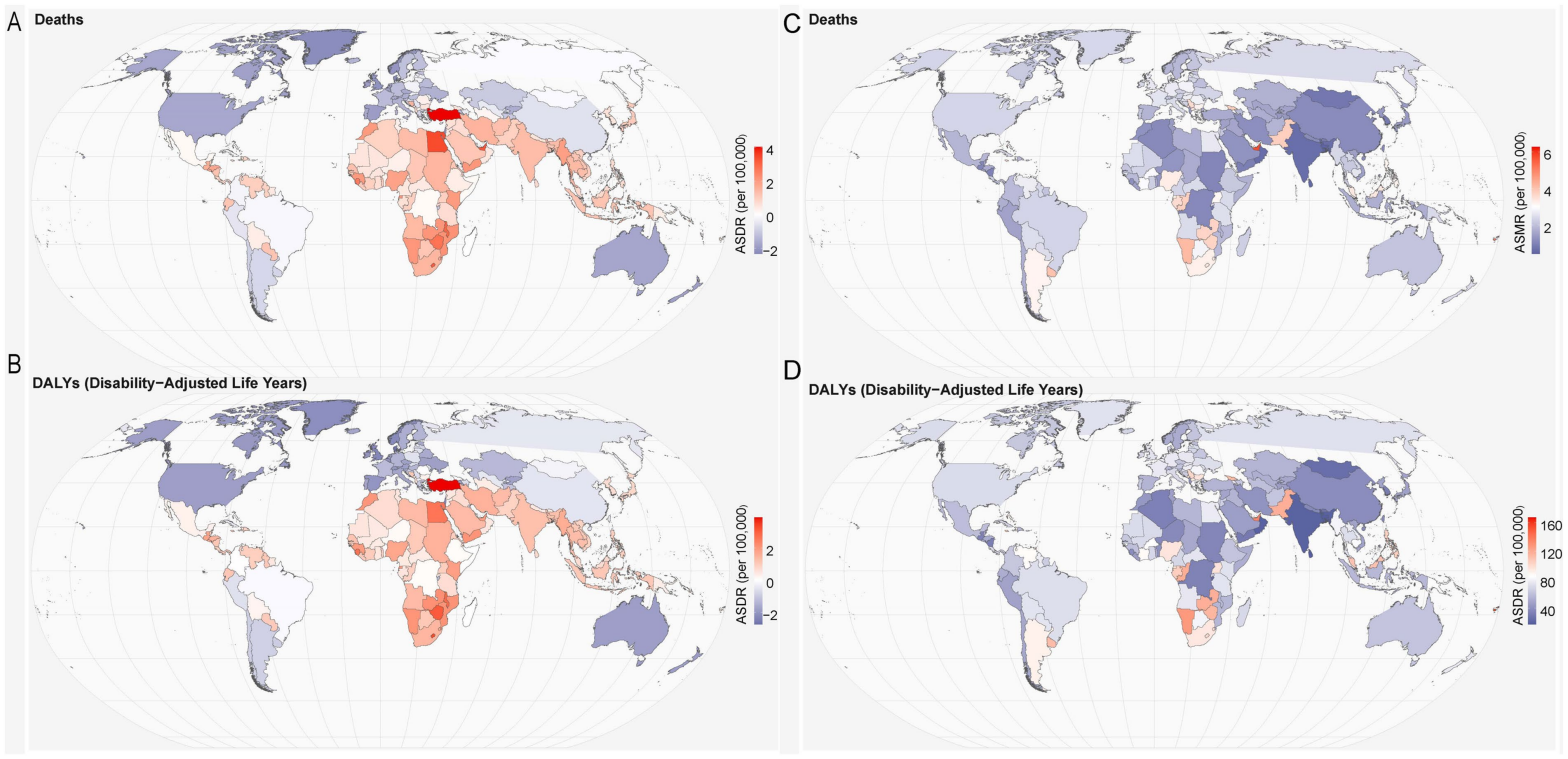
The spatial distribution of breast cancer Age-standardized mortality rate (ASMR) **(A)** and Age-standardized DALY rate ASDR **(B)** attributable to high red meat consumption, and the EAPC in breast cancer ASMR **(C)** and ASDR **(D)** attributable to high red meat consumption. **(A)** ASMR per 100,000 people in 2021. **(B)** ASDR per 100,000 people in 2021. **(C)** Estimated annual percentage change (EAPC) in ASMR from 1990 to 2021. **(D)** EAPC in ASDR from 1990 to 2021.

As shown in Figure 2A, countries like Turkey and Egypt exhibit increasing ASDR for breast cancer, while Western Europe shows declining trends, indicating a reduction in breast cancer mortality in those regions. Figure 2B reflects similar trends for DALYs, Turkey and certain African countries experiencing a growing burden of breast cancer, while blue-shaded areas, including parts of Europe, demonstrate a decrease in DALYs. In Figure 2C, countries such as American Samoa and India are highlighted as having the highest ASMR per 100,000 individuals, underscoring the disproportionate impact of breast cancer in these regions. Meanwhile, Figure 2D shows that the DALYs burden in countries like India and Nigeria is significantly higher, emphasizing the long-term health impact of breast cancer in these nations. Furthermore, analysis of EAPC in breast cancer deaths and DALYs between 1990 and 2021 revealed increasing trends in many countries. China’s EAPC for breast cancer deaths was 1.26 (95% CI: 1.12–1.4), while the EAPC for DALYs was 1.31 (95% CI: 1.16–1.45). The United States and India reported EAPCs of 0.74 and 0.9, respectively, showing a slower, yet still ongoing, rise in breast cancer burden.

### 3.3 Global trends in breast cancer attributable to a diet high in red meat by SDI and age

**Figure 3:**
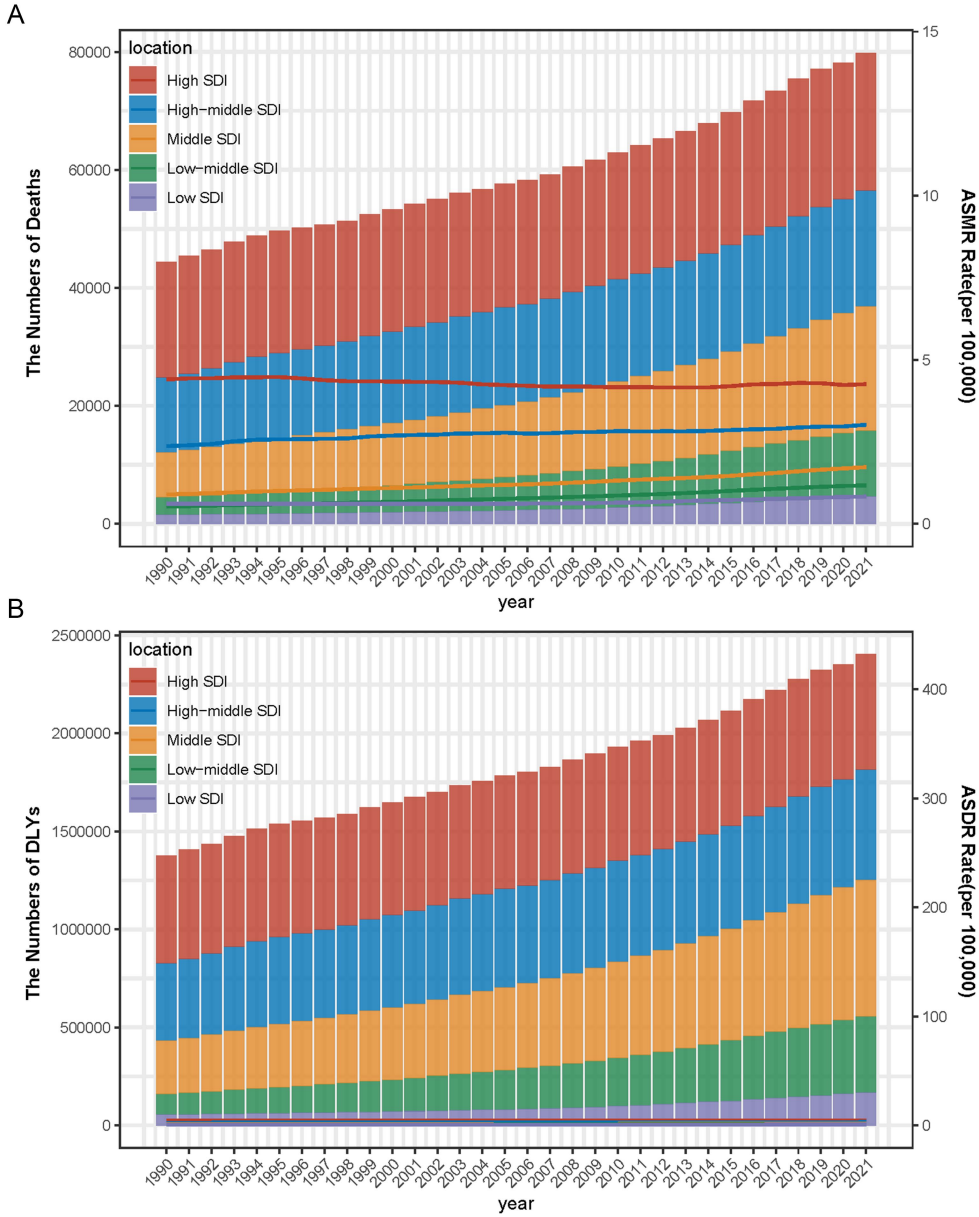
Number and rate of breast cancer deaths **(A)** and DALYs **(B)** are attributable to a diet high in red meat from 1990 to 2021 by SDI level. The bars represent the number of breast cancer deaths **(A)** and DALYs **(B)** attributable to a diet high in red meat from 1990 to 2021, colored by SDI level. The line represents the mean ASMR **(A)** and ASDR **(B)** (per 100,000) attributable to a diet high in red meat at the global level. The shaded area represents the 95% UI for the mean rate.

Globally, the burden of breast cancer attributable to a diet high in red meat has steadily increased across all SDI regions from 1990 to 2021. In high SDI regions, breast cancer-related deaths rose from approximately 40,000 deaths in 1990 to nearly 80,000 deaths in 2021, while high-middle SDI regions experienced an increase from around 25,000 deaths in 1990 to over 60,000 deaths in 2021. Low SDI and low-middle SDI regions, although reporting lower absolute numbers of deaths, with 5,000 and 10,000 deaths, respectively, in 2021, still experienced a significant relative increase compared to 1990 (Figure 3A). In terms of DALYs, a similar upward trend can be observed, with high SDI regions seeing an increase from 1 million DALYs in 1990 to over 2 million DALYs by 2021, while high-middle SDI regions grew from 1 million to nearly 2 million DALYs. Low-middle SDI and low SDI regions also experienced growth in DALYs, with numbers rising from 200,000 to over 500,000 in low-middle SDI regions and from 100,000 to 300,000 in low SDI regions (Figure 3B).

**Figure 4:**
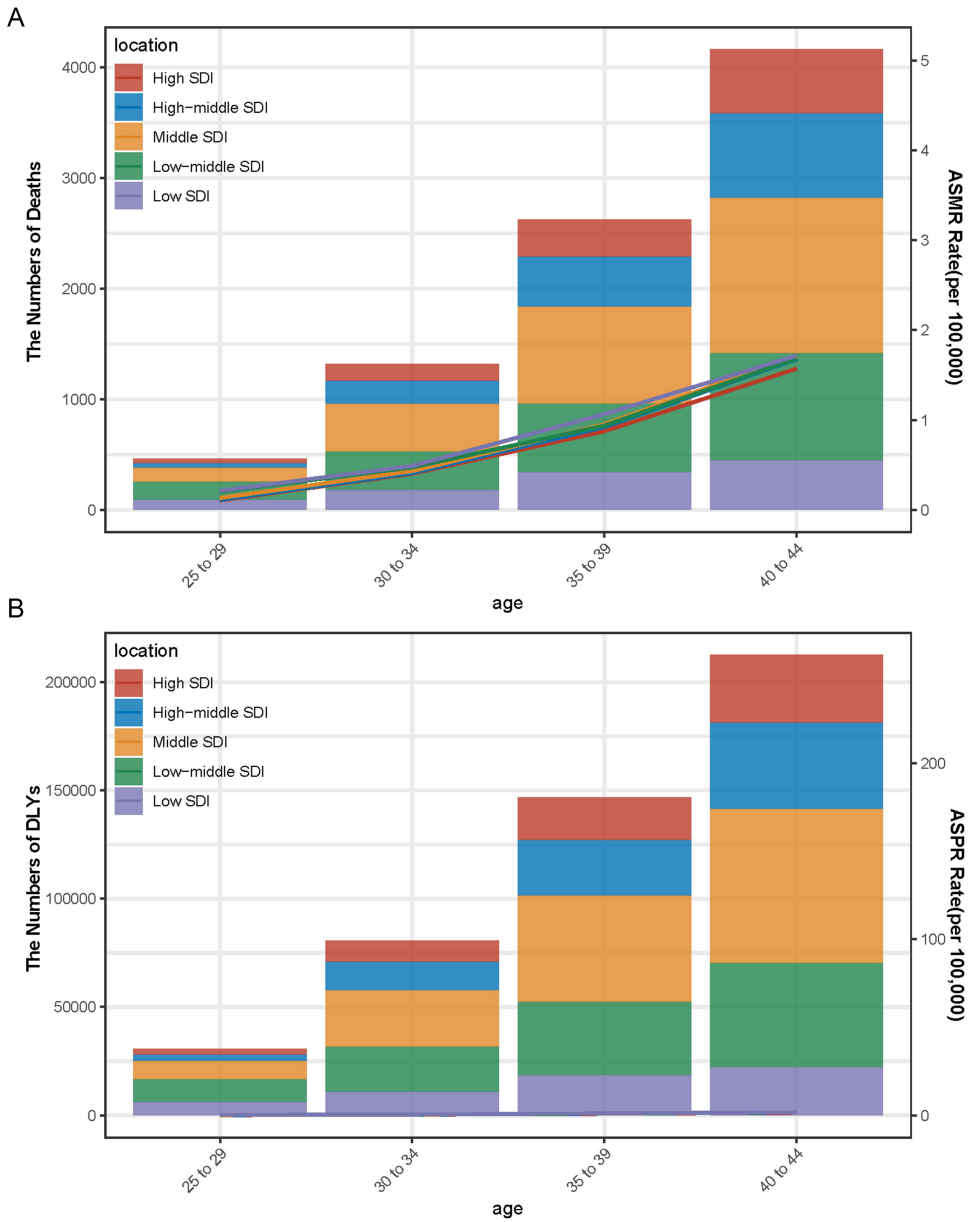
Number and rate of breast cancer deaths **(A)** and DALYs **(B)** attributable to a diet high in red meat by age and SDI level in 2021. The bars represent the number of breast cancer deaths **(A)** and DALYs **(B)** attributable to a diet high in red meat in 2021, colored by SDI level. The line represents the mean ASMR **(A)** and ASDR **(B)** (per 100,000) attributable to a diet high in red meat at the global level.

When examining age-specific trends for 2021, the 40–44 age group had the highest number of breast cancer-related deaths across all SDI levels. High SDI regions reported over 4,000 deaths, followed by high-middle SDI regions with around 3,000 deaths, middle SDI regions with 2,500 deaths, low-middle SDI regions with just under 2,000 deaths, and low SDI regions with fewer than 1,000 deaths (Figure 4A). Similarly, DALYs peaked in the 40–44 age group, with high SDI regions reporting more than 200,000 DALYs, high-middle SDI regions nearing 150,000 DALYs, middle SDI regions approaching 120,000 DALYs, low-middle SDI regions exceeding 100,000 DALYs, and low SDI regions reporting fewer than 50,000 DALYs (Figure 4B).

**Figure 5:**
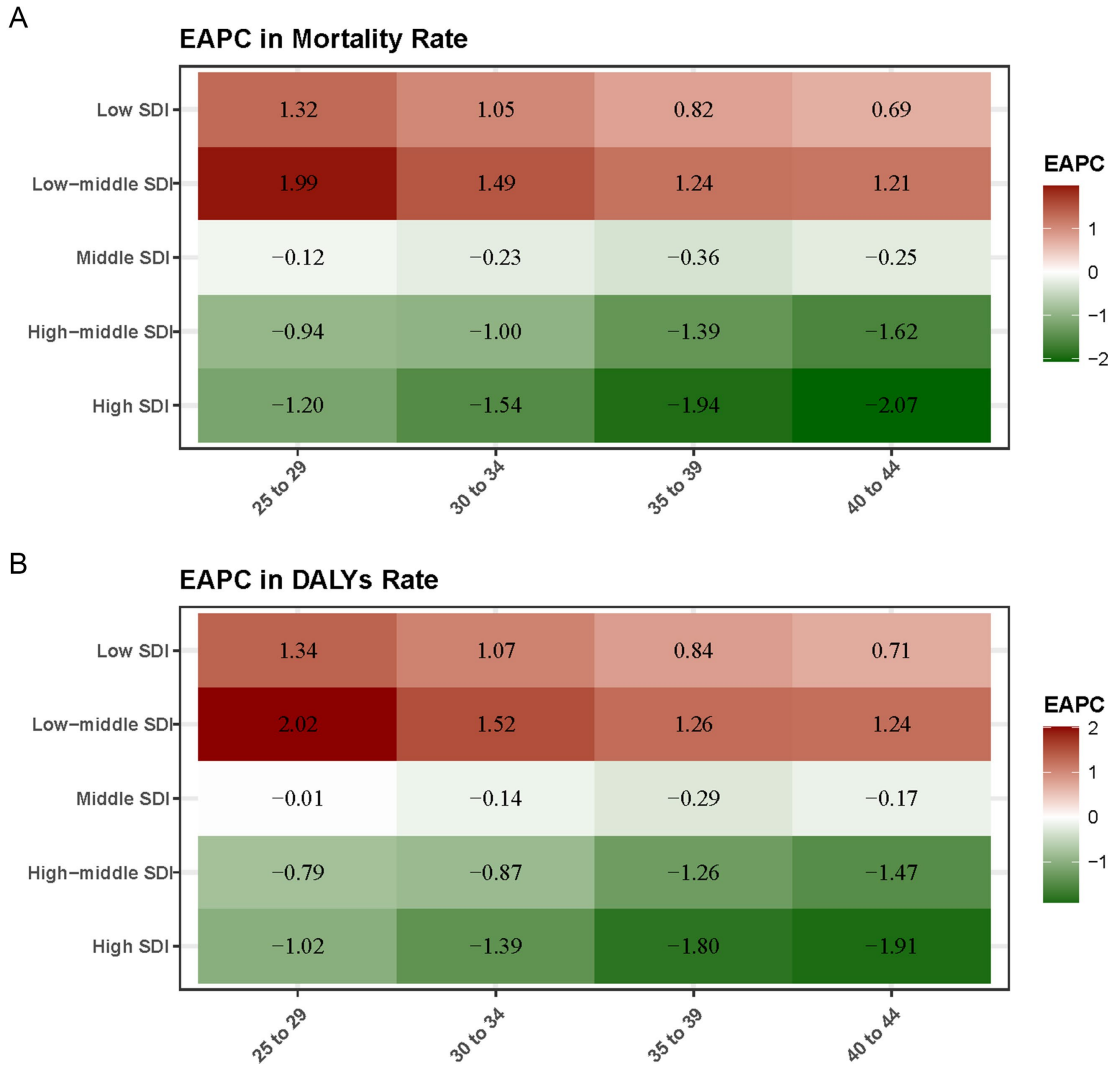
Average annual percentage change (EAPC) in breast cancer mortality rate **(A)** and DALYs rate **(B)** attributable to a diet high in red meat from 1990 to 2021 by SDI level and age. The heatmaps represent the EAPC in breast cancer mortality **(A)** and DALYs **(B)** attributable to a diet high in red meat from 1990 to 2021, colored by SDI level and age.

Between 1990 and 2021, low-middle SDI regions showed the highest growth in mortality rates, with an EAPC of 1.99% in the 25–29 age group, 1.49% in the 30–34 age group, and 1.21% in the 40–44 age group. In contrast, high SDI regions saw consistent decreases in mortality, with a-1.54% reduction in the 30–34 age group and a − 2.07% reduction in the 40–44 age group (Figure 5A). For DALYs, the low-middle SDI regions again showed the largest increases, with an EAPC of 2.02% in the 25–29 age group, 1.52% in the 30–34 age group, and 1.24% in the 40–44 age group. High SDI regions saw declines in DALYs, with the largest reduction of −1.91% occurring in the 40–44 age group (Figure 5B).

### 3.4 Factors associated with the burden of breast cancer attributable to a diet high in red meat

**Figure 6:**
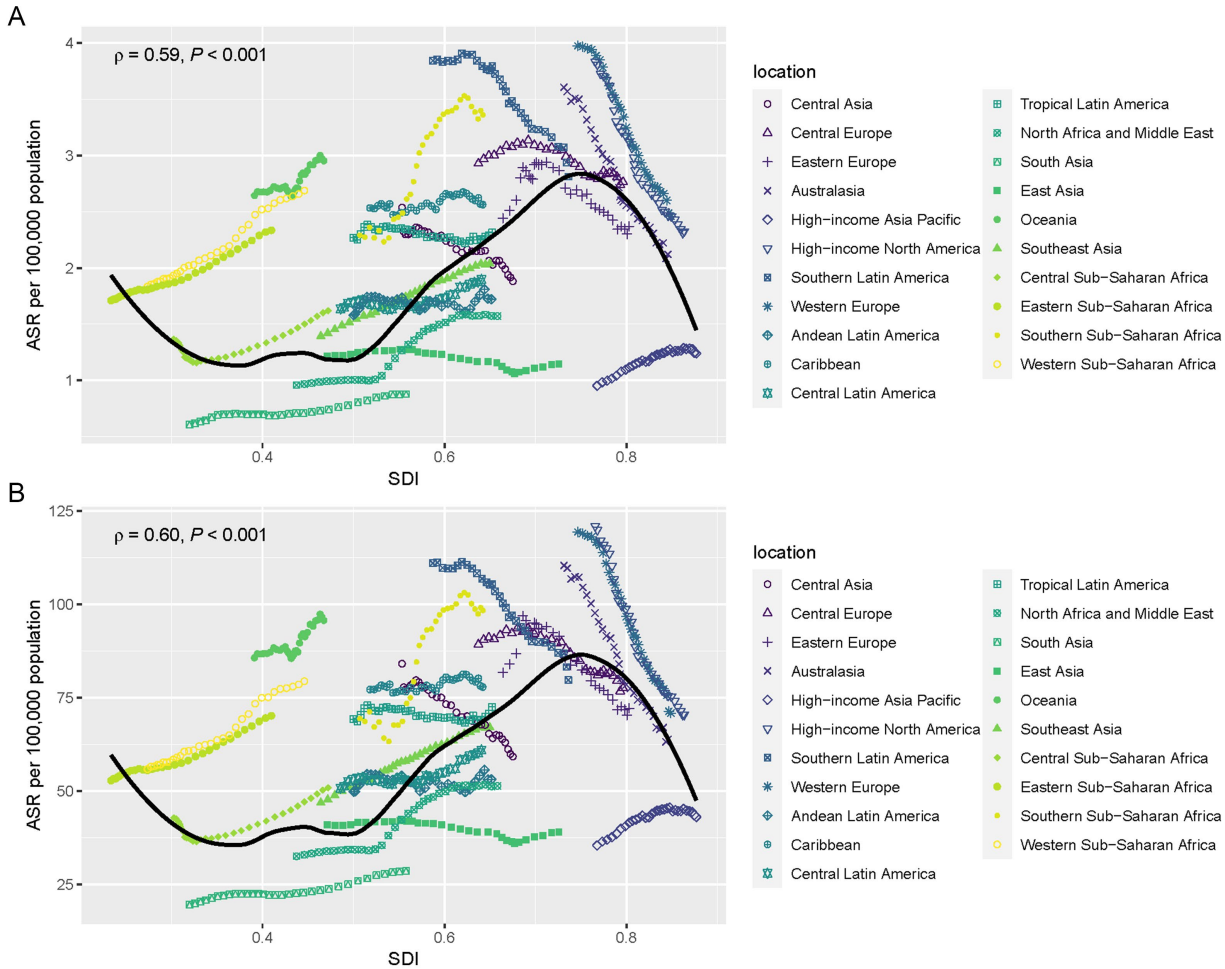
ASMR **(A)** and ASDR **(B)** for breast cancer is attributable to a diet high in red meat by SDI level in 2021. The scatter plots show the relationship between ASMR **(A)** and ASDR **(B)** and SDI for various regions. The line represents the global trend, showing a nonlinear “S”-shaped association between SDI and the burden of breast cancer. High SDI regions, such as High-income North America and Western Europe, have the highest ASMR and ASDR, while low SDI regions, such as South Asia and Eastern Sub-Saharan Africa, show the lowest rates.

Overall, there was a nonlinear “S”-shaped association between the overall ASMR and SDI, with ASMR progressively increasing when the SDI was less than 0.45, and then decreasing as the SDI rose beyond 0.75. Among the different regions, the highest ASMR related to a diet high in red meat was observed in High-income North America and Western Europe, followed by Eastern Europe and Central Asia, while the lowest ASMR occurred in South Asia and Eastern Sub-Saharan Africa (Figure 6A). A similar association was observed between ASDR and SDI, with ASDR rising rapidly in regions with higher SDI, particularly in High-income North America and Western Europe (Figure 6B).

**Figure 7:**
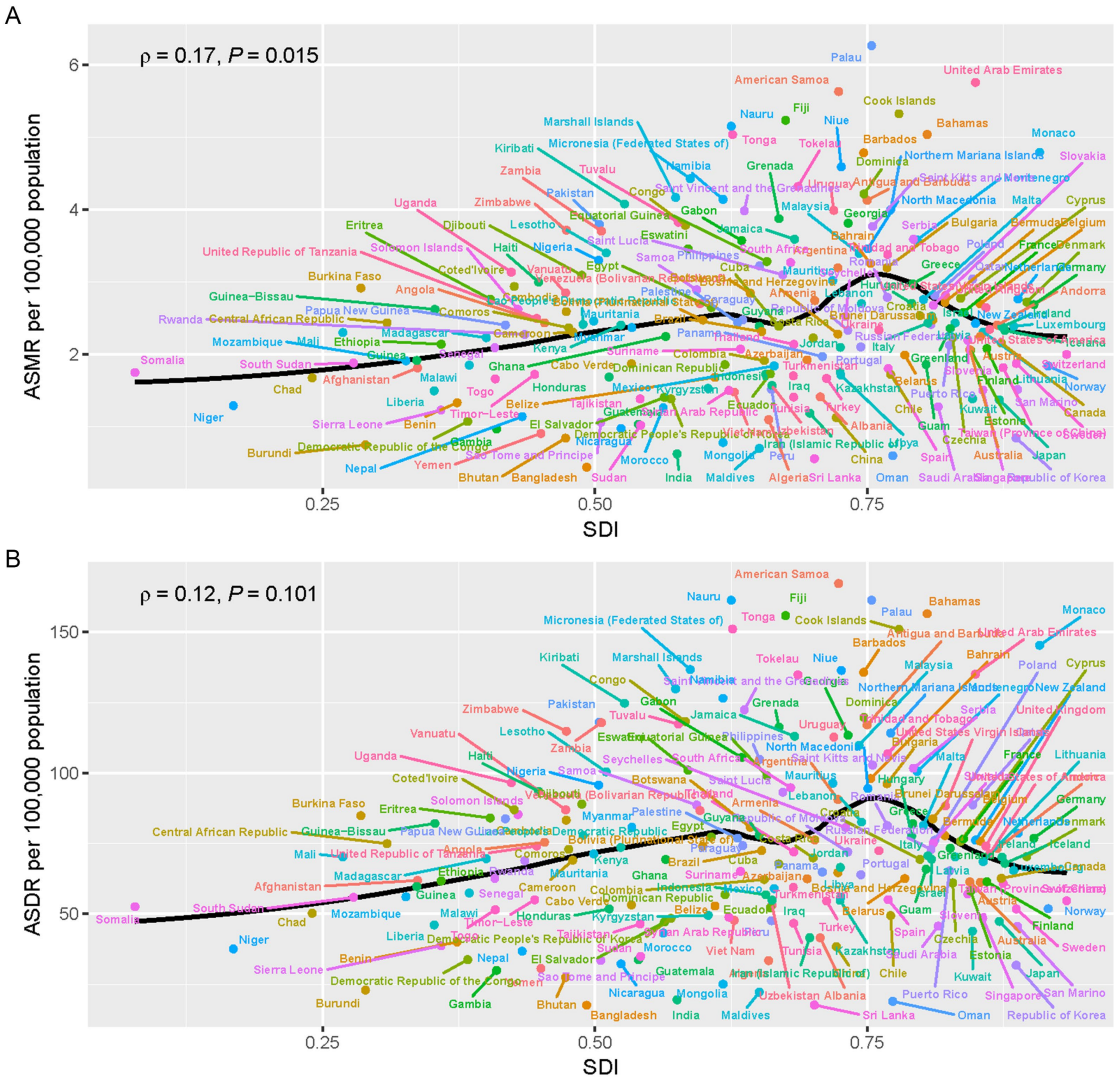
The relationship between SDI and breast cancer burden attributable to a diet high in red meat across 204 countries and territories in 2021. **(A)** Shows the relationship between ASMR and SDI, while **(B)** shows the relationship between ASDR and SDI. The scatter plots highlight those countries with moderate SDI, such as those in Eastern Europe and Central Asia, experience the highest ASMR and ASDR, while countries with both low and high SDI, such as South Asia and Australasia, have the lowest rates.

In 2021, across 204 countries and territories globally, the relationships between ASMR and ASDR attributable to breast cancer and SDI followed an initial increase before decreasing as SDI continued to rise. The highest ASMR and ASDR were recorded in countries with moderate SDI, such as Eastern Europe and Central Asia, while the lowest rates were seen in countries with both low and high SDI, such as South Asia and Australasia (Figures 7A,B). The nonlinear relationship between breast cancer burden and SDI highlights the complexity of this disease’s global distribution and the various factors contributing to its risk across diverse regions.

## 4 Discussion

In our study, we presented the spatiotemporal trends breast cancer in adult women and its associated risk with diet in high red meat over the past 32 years based on the latest GBD 2021 study. The selection of women aged 25–45 in breast cancer research is based on several factors. First, this age group often exhibits distinct biological characteristics, including denser breast tissue, which can complicate early detection (16). Additionally, younger women are more likely to develop aggressive subtypes such as triple-negative breast cancer, which is harder to treat and has a worse prognosis (17). Moreover, adult women are more likely to experience hormonal fluctuations and pregnancies, etc. which may influence the incidence and progression of breast cancer (18, 19). Furthermore, this group is at a crucial intersection of lifestyle choices and health outcomes, making dietary investigation particularly salient. The processed meats are also widely recognized as a cancer risk factor. However, we only focused on unprocessed red meat due to following reasons. (1) Processed meat is a group 1 (carcinogens) while red meat is group 2A carcinogen (20), therefore, its association with breast cancer remains controversial. (2) The carcinogenic mechanisms and risk levels of processed meats and unprocessed red meat differ, and separating these two categories allows for a more precise evaluation of their respective impacts (20). (3) The risk assessment in the GBD 2021 study was also conducted separately for these two types of meat, and our research design follows this methodological framework. Our analysis identifies a significant relationship between high red meat consumption and increased breast cancer risk in this population, underscoring the importance of dietary interventions, which aligns with previous studies (2, 21).

The study shows that breast cancer-related deaths and DALYs increased significantly, with nearly 80,000 deaths linked to red meat consumption in 2021. This trend highlights the serious public health concerns related to dietary choices, especially across different socio-economic regions. High-SDI regions have reduced ASMR and ASDR, indicating successful healthcare improvements. In contrast, high-middle and low-middle SDI regions, particularly in Southern Sub-Saharan Africa, have seen significant increases in these metrics, pointing to an urgent need for targeted intervention. Regionally, East Asia has the highest breast cancer-related deaths, while Australasia has seen reductions. The high mortality rates in Central Sub-Saharan Africa and South Asia suggest ongoing dietary risk issues that require further research and action. Overall, despite global health advancements, disparities in dietary-related breast cancer persist. The study also highlights the significant global burden of breast cancer linked to high red meat consumption, revealing marked disparities across countries and regions. In 2021, China reported the highest number of breast cancer deaths attributed to red meat, reflecting a troubling increase since 1990. This rise is associated with dietary shifts, urbanization, obesity, and sedentary lifestyles, exacerbated by insufficient public health measures (22). India also faces a serious breast cancer burden, with over 190 deaths and nearly 6,900 DALYs related to red meat consumption, indicating a pressing need for targeted public health strategies. Smaller island nations, including American Samoa, Nauru, and Palau, show particularly high ASDRs exceeding 161 per 100,000, pointing to vulnerabilities linked to dietary practices and healthcare access.

Overall, these data, along with the mapped geographical distributions, indicate that high red-meat consumption has become a significant factor contributing to the global breast cancer burden, particularly in low- and middle-income countries. As these nations continue to develop economically and adopt lifestyle changes, the link between breast cancer incidence, mortality, and unhealthy dietary habits is becoming increasingly evident. This data emphasizes the need for better dietary education and cancer prevention strategies, particularly in areas with high red meat consumption (23). Consequently, it is essential to implement widespread awareness and preventive measures in high-burden countries and regions, promoting healthier dietary habits to mitigate future breast cancer risks. Future research should focus on understanding these disparities and evaluating interventions to reduce breast cancer linked to diet (24).

High and high-middle SDI regions have experienced significant absolute increases in breast cancer deaths and DALYs from 1990 to 2021, driven by population aging, higher red meat consumption, and improved detection, contributing to the largest global burden. However, high SDI regions have seen decreases in mortality and DALYs in younger age groups due to advancements in screening, early diagnosis, and treatment. In contrast, low and low-middle SDI regions have seen steep relative increases in breast cancer deaths and DALYs, particularly among younger women, due to delayed diagnosis, limited healthcare resources, and rising lifestyle-related risk factors linked to urbanization and globalization (25).

Mortality and DALYs peak in the 40–44 age group across all SDI regions, highlighting the need for targeted interventions for adult women. The greater burden in higher SDI regions suggests a stronger link to dietary and lifestyle factors like red meat consumption, whereas the poorer outcomes in lower SDI regions underline the need for improved access to early diagnosis and treatment. Preventive strategies, including dietary education and public health campaigns to reduce red meat consumption, are crucial globally. High SDI regions should focus on continued dietary reform and innovations in care, while low and low-middle SDI regions must prioritize building healthcare infrastructure, raising awareness, and addressing modifiable risk factors. Tailoring strategies to regional and cultural contexts is essential for reducing disparities in the global breast cancer burden (26).

### 4.1 Strengths and limitations

This GBD-based study is the most comprehensive attempt to reveal global breast cancer mortality rates attributed to red meat consumption. The findings enhance evidence on global breast cancer risks and the associated burden, which is vital for prevention and management. However, there are limitations. Some regions lack cancer registries, causing data gaps that affect estimates. Additionally, GBD 2021 considers only a few behavioral and metabolic risks, which are essential for a complete assessment of breast cancer burden (27). This study only discusses DALYs, and mortality rates and does not focus on breast cancer incidence and prevalence. Moreover, the impact of other dietary and lifestyle factors was not analyzed, and the complex interaction between these and red meat consumption needs further investigation. Future research should aim to assess the synergistic effects of diet, physical activity, and other environmental factors in contributing to breast cancer incidence and outcomes. Region-specific research is also crucial to developing culturally sensitive interventions tailored to local dietary practices (26). Data sharing improves research integrity and transparency, supporting peer validation and deeper exploration of findings. It is particularly important for developing countries. Our study is significant because all data is freely available for download, providing detailed estimates of the global breast cancer burden. In the future, policymakers can use this information for better resource allocation and public health strategies. However, our results might reflect outdated data on the disease burden, and predicting future trends requires more recent information.

## 5 Conclusion

The study concludes that the global burden of breast cancer linked to red meat consumption is steadily increasing, driven by the globalization of Western dietary patterns and persistent healthcare disparities across regions. While high-SDI regions continue to bear the largest absolute burden, the rising relative increases in low- and lower-middle-SDI regions highlight significant emerging challenges that require immediate action. These trends are exacerbated by economic development and lifestyle transitions in low- and middle-income countries, which are contributing to the growing breast cancer burden in these regions. Strengthening early detection and prevention strategies, particularly those focusing on modifiable risk factors like diet, will be essential to mitigating the global impact of breast cancer in the coming decades. High-SDI countries have made notable progress due to advancements in healthcare systems and prevention programs, yet these efforts must be adapted and expanded to address the unique challenges faced by low- and middle-SDI regions. This study underscores the urgent need for targeted interventions, such as dietary modifications and improved access to healthcare, to reduce breast cancer risks in high-burden areas. By addressing healthcare disparities and incorporating dietary interventions into broader cancer control strategies, significant progress can be made in reducing breast cancer-related deaths and disability worldwide.

## Data Availability

The original contributions presented in the study are included in the article/Supplementary material, further inquiries can be directed to the corresponding author.

## Footnotes

^1^ https://vizhub.healthdata.org/gbd-results/

^2^ https://eatforum.org/eat-lancet-commission/

^3^ https://www.r-project.org/
