# Supplementary material for: Global, regional, and national analyses of the burden among adult women of breast cancer attributable to diet high in red meat from 1990 to 2021: longitudinal observational study
Source: Front Public Health. 2025 May 13;13:1580177. doi: 10.3389/fpubh.2025.1580177 (PMC12107595; doi:10.3389/fpubh.2025.1580177)
Supplement: Supplementary file 1 [file Table_1.docx]

| **Supplementary Table 1.** Top 10 countries or territories with the highest number of breast cancer Deaths related to diet high in red meat in 2021. | |
| --- | --- |
| **Location** | **No. (95% UI)** |
| China | 12074.64 (26711--6.43) |
| United States of America | 7227.01(15498--3.09) |
| India | 3947.51(9023--0.07) |
| Russian Federation | 3417.07(7194--1.56) |
| Brazil | 3200.67(6859--1.91) |
| Germany | 2828.07(6078--1.51) |
| Pakistan | 2535.19(5633--0.74) |
| Indonesia | 2395.7(6239--0.1) |
| Japan | 2208.65(4803--0.58) |
| France | 2067.92(4412--0.72) |

UI: uncertainty interval. The above data has been adjusted by DisMod MR version 2.1.
