# Supplementary material for: Global, regional, and national analyses of the burden among adult women of breast cancer attributable to diet high in red meat from 1990 to 2021: longitudinal observational study
Source: Front Public Health. 2025 May 13;13:1580177. doi: 10.3389/fpubh.2025.1580177 (PMC12107595; doi:10.3389/fpubh.2025.1580177)
Supplement: Supplementary file 2 [file Table_2.docx]

| **Supplementary Table 2** Top 10 countries or territories with the highest number of breast cancer DALYs related to diet high in red meat in 2021. | |
| --- | --- |
| **Location** | **No. (95% UI)** |
| China | 399987.95(889529--235.05) |
| United States of America | 192353.67(412165--87.65) |
| India | 130243.19(300205--2.51) |
| Brazil | 99470.13(211810--57.2) |
| Russian Federation | 93415.67(195930--40.49) |
| Pakistan | 92230.51(206035--28.19) |
| Indonesia | 85577.11(221185--3.77) |
| Germany | 64065.17(137320--33.57) |
| Nigeria | 60658.61(140022--7.19) |
| Japan | 56752.27(123318--15.53) |

DALYs: disability-adjusted life-years. UI: uncertainty interval. The above data has been adjusted by DisMod MR version 2.1.
