# Supplementary material for: Global, regional, and national analyses of the burden among adult women of breast cancer attributable to diet high in red meat from 1990 to 2021: longitudinal observational study
Source: Front Public Health. 2025 May 13;13:1580177. doi: 10.3389/fpubh.2025.1580177 (PMC12107595; doi:10.3389/fpubh.2025.1580177)
Supplement: Supplementary file 3 [file Table_3.docx]

| **Supplementary Table 3.** Top 10 countries or territories with the highest breast cancer ASDR (per 100 000) attributable to diet high in red meat in 2021. | |
| --- | --- |
| **Location** | **No. (95% UI)** |
| American Samoa | 167.25(377--0.07) |
| Nauru | 161.33(419--0.08) |
| Palau | 161.32(356--0.07) |
| Bahamas | 156.56(345--0.09) |
| Fiji | 155.84(342--0.09) |
| Tonga | 151.1(339--0.06) |
| Cook Islands | 150.99(346--0.08) |
| Monaco | 145.33(327--0.09) |
| Micronesia (Federated States of) | 136.72(321--0.06 |
| Niue | 136.41(310--0.05) |

ASDR: age-standardized DALYs rate. UI: uncertainty interval. The above data has been adjusted by DisMod MR version 2.1.
