# Supplementary material for: Global, regional, and national analyses of the burden among adult women of breast cancer attributable to diet high in red meat from 1990 to 2021: longitudinal observational study
Source: Front Public Health. 2025 May 13;13:1580177. doi: 10.3389/fpubh.2025.1580177 (PMC12107595; doi:10.3389/fpubh.2025.1580177)
Supplement: Supplementary file 4 [file Table_4.docx]

| **Supplementary Table 5.** Top 10 countries or territories with the highest or lowest EAPC in the ASMR (per 100 000) attributable to diet high in red meat,1990–2021. | |
| --- | --- |
| **Location** | **No. (95% CI)** |
| Turkey | 4.02 (3.26-4.79) |
| Egypt | 3.4(2.94-3.87) |
| United Arab Emirates | 3.24(2.81-3.68) |
| Lesotho | 3.15(2.73-3.57) |
| Malawi | 2.99(2.88-3.09) |
| Zimbabwe | 2.77(2.09-3.45) |
| Sierra Leone | 2.58(2.38-2.78) |
| Sao Tome and Principe | 2.19(2.05-2.33) |
| Mozambique | 2.17(2.09-2.25) |
| Equatorial Guinea | 2.16(2.05-2.27) |
| Denmark | -2.33(-2.43--2.23) |
| Bermuda | -2.3(-2.53--2.07) |
| Greenland | -2.26(-2.41--2.11) |
| United Kingdom | -2.14(-2.21--2.06) |
| Norway | -2.08(-2.21--1.94) |
| Malta | -2.05(-2.2--1.91) |
| Israel | -2.04(-2.26--1.83) |
| Spain | -1.94(-2--1.88) |
| Ireland | -1.91(-2.01--1.81) |
| Netherlands | -1.9(-2.02--1.78) |

ASMR: age-standardized mortality rate. EAPC: estimated annual percentage change.CI: confidence interval.The above data has been adjusted by DisMod MR version 2.1.
